# Mitochondrial DNA leakage triggers inflammation in age-related cardiovascular diseases

**DOI:** 10.3389/fcell.2024.1287447

**Published:** 2024-02-15

**Authors:** Wanyue Ding, Jingyu Chen, Lei Zhao, Shuang Wu, Xiaomei Chen, Hong Chen

**Affiliations:** ^1^ Heilongjiang Academy of Traditional Chinese Medicine, Harbin, China; ^2^ Department of Chinese Medicine Internal Medicine, Xiyuan Hospital, China Academy of Chinese Medical Sciences, Beijing, China; ^3^ Southern Medical University Affiliated Qiqihar Hospital, The First Hospital of Qiqihar, Qiqihaer, Heilongjiang, China; ^4^ Integrated Traditional Chinese and Western Medicine Syndrome Laboratory, School of Traditional Chinese Medicine, Southern Medical University, Guangzhou, Guangdong, China

**Keywords:** cardiovasuclar diseases, inflammation, mitochondrial DNA, senescence, innate immunity

## Abstract

Mitochondrial dysfunction is one of the hallmarks of cardiovascular aging. The leakage of mitochondrial DNA (mtDNA) is increased in senescent cells, which are resistant to programmed cell death such as apoptosis. Due to its similarity to prokaryotic DNA, mtDNA could be recognized by cellular DNA sensors and trigger innate immune responses, resulting in chronic inflammatory conditions during aging. The mechanisms include cGAS-STING signaling, TLR-9 and inflammasomes activation. Mitochondrial quality controls such as mitophagy could prevent mitochondria from triggering harmful inflammatory responses, but when this homeostasis is out of balance, mtDNA-induced inflammation could become pathogenic and contribute to age-related cardiovascular diseases. Here, we summarize recent studies on mechanisms by which mtDNA promotes inflammation and aging-related cardiovascular diseases, and discuss the potential value of mtDNA in early screening and as therapeutic targets.

## 1 Introduction

Cardiovascular diseases have long been the leading cause of death. It is estimated that there were 620 million patients with cardiovascular diseases worldwide in 2021, along with more than 20 million cardiovascular deaths (Lindstrom et al., 2022). Population aging has become an important factor worldwide contributing to the increasing burden of cardiovascular diseases. Age has long been identified as a significant risk factor for cardiovascular events. According to the statistics from American Heart Association, the overall prevalence of cardiovascular diseases (including coronary heart disease, heart failure, stroke, hypertension) in adults is approximately 48%, but it reaches around 78% in the population aged 60 to 79, and rises to about 90% in those aged 80 and above (Benjamin et al., 2019). Additionally, traditional risk factors such as hyperlipidemia and diabetes become more prevalent with advancing age. The normal physiology of the cardiovascular system is also significantly influenced by aging, including myocardial hypertrophy, increased arterial stiffness, and impaired endothelial function (Lakatta and Levy, 2003a; Lakatta and Levy, 2003b). Many physiological changes during the aging process contribute to impairment in cardiovascular system, among which age-related systemic chronic inflammation playing a significant role (Lettino et al., 2022).

Aging is characterized by systemic chronic inflammation, or described as “inflamm-aging,” which is manifested by senescent cells secreting inflammatory factors in the absence of acute infection, which also induces the senescence of normal cells in a paracrine manner (Li et al., 2023). The burden of senescent cells increases due to the weakened clearance of senescent cells, thus forming a vicious cycle of inflammation and aging, leading to cardiac and vascular dysfunction (Liberale et al., 2020). Inappropriate activation of chronic inflammation during aging involves complex mechanisms. Low-level toxin exposure derived from dysregulated microbiota and chronic infections (such as periodontitis) may participate in this process. Cellular senescence mechanisms also considered to play a key role, including telomere shortening, genome instability, protein catabolism defects, autophagy and mitophagy dysregulation, and mitochondrial dysfunction (Sanada et al., 2018).

The cardiovascular system is composed of terminally differentiated cells such as cardiomyocytes and vascular smooth muscle cells (VSMCs), which are rich in mitochondria and maintain the circulatory function under continuous metabolic and mechanical stress. Mitochondria is essential for maintaining the function of cardiovascular system (Abdellatif et al., 2023). Mitochondrial dysfunction is considered one of the hallmarks of cardiovascular aging. In addition to its functions in energy metabolism, mitochondria also maintain intracellular calcium homeostasis, redox balance, and act as a signaling center to regulate cellular behaviors such as mitophagy and apoptosis (Galluzzi et al., 2012).

There is a close connection between mitochondrial dysfunction and inflammation. As an organelle similar to prokaryotes, some structural components of mitochondria could directly induce inflammatory response as damage-associated molecular patterns (DAMPs) (Marchi et al., 2023). Mitochondrial DNA (mtDNA) with a circular double-stranded structure, can be leaked into the cytoplasm as a result of mitochondrial damage, and is recognized by pattern recognition receptors and exogenous DNA receptors, initiating inflammatory response (Wein and Sorek, 2022). Although this helps to clear injured cells to maintain tissue homeostasis, recent studies also suggested that it involved in aging-related cardiovascular diseases (Galluzzi et al., 2018; Harapas et al., 2022).

This review aims to summarize the mechanisms mtDNA initiating inflammatory responses and its role in aging-related cardiovascular diseases, and discusses potential therapeutic strategies of inhibiting mtDNA-mediated inflammation for aging-related cardiovascular diseases.

## 2 Mechanisms of mitochondrial DNA leakage triggered inflammation

mtDNA is a small double-stranded circular molecule with a full length of 16,569 base pairs. It only includes 13 genes encoding oxidative phosphorylation-related proteins, ribosomal RNAs and transfer RNAs. It has also been reported in recent studies that mtDNA could be transcribed into non-coding RNAs (Gao et al., 2018). The remaining mitochondrial proteins rely on nuclear gene expression and then imported into the mitochondria (Calvo and Mootha, 2010). Since mitochondria are the site of reactive oxygen species (ROS) generation, mtDNA in a bare nucleoid form is susceptible to damage and accumulates with aging (Gredilla et al., 2010). Under physiological conditions, damaged mitochondria are cleared through mitochondrial quality control such as mitochondrial fission, fusion, and mitophagy (Picca et al., 2018). But when these mechanisms fail or mitochondria are irreversibly damaged, mtDNA will leak from the mitochondrial matrix and be recognized as DAMPs to trigger inflammatory response (Liu et al., 2022).

Although the mechanism of how mtDNA leaks into the cytoplasm or extracellular space has not been fully elucidated, the following mechanisms are considered to be involved. The mitochondrial permeability transition pore (mPTP) is a protein pore complex located at the contact point between the inner and outer membranes of mitochondria. The open of mPTP could result in the leakage of mtDNA into the cytoplasm (Yu et al., 2020). During apoptosis, BCL-2 Associated X (BAX) and BCL-2 Homologous Antagonist/Killer (BAK) also interact with mPTP subunits to regulate the open of mPTP (Rongvaux et al., 2014; White et al., 2014). Activated caspase-1 also involves in mitochondrial pore formation through activating the pore-forming protein gasdermin D (Huang et al., 2020). In addition, mitochondria-derived vesicles (MDVs) can also selectively regulate the transfer of damaged mitochondrial contents to lysosomes for degrading (Todkar et al., 2021). However, it is also reported that circulating mtDNA-containing MDVs could cause inflammatory responses (Picca et al., 2020).

### 2.1 cGAS-STING pathway

Since the DNA of eukaryotic cells mainly locates in the nucleus or mitochondria, the DNA in the cytoplasm is usually recognized as exogenous from pathogens to initiate innate immune response (Civril et al., 2013). However, mtDNA leaking from mitochondria and accumulating in the cytoplasm, could also be recognized as powerful stimulator of inflammatory response through similar pathways (West et al., 2011). Type I interferon (IFN) response is an important signaling pathway against pathogenic infection, and Stimulator of Interferon Genes (STING) was identified as a protein mediating type I IFN response first (Ishikawa and Barber, 2008; Zhong et al., 2008; Sun et al., 2009). Although STING could be activated by cytoplasmic DNA, subsequent studies revealed that the ligands of STING are cyclic dinucleotides rather than cytoplasmic DNA, including cyclic diadenosine monophosphate (c-dAMP) and cyclic diguanylate monophosphate (c-dGMP) (Burdette et al., 2011). Cyclic GMP-AMP Synthase (cGAS) is the upstream DNA sensor of STING. In the presence of ATP and GTP, cGAS binds to and recognizes cytoplasmic DNA to catalyze the production of cyclic guanosine monophosphate-adenosine monophosphate (cGAMP), which could activate STING as its ligand (Sun et al., 2013; Wu et al., 2013).

In short, when mtDNA leaks into the cytoplasm, cGAS binds to mtDNA and catalyzes the synthesis of cGAMP. cGAMP acts as a ligand for STING to cause conformational changes and activate it (De Gaetano et al., 2021). The activated STING is transferred from the endoplasmic reticulum to the Golgi apparatus and recruited TANK Binding Kinase 1 (TBK1) and Inhibitor of Nuclear Transcription Factor-κB Kinase (IKK). Activated TBK1 and IKK phosphorylate downstream Interferon Regulatory Factor 3 (IRF3) and Inhibitor α of Nuclear Transcription Factor-κB (IκBα) respectively, following by activated IRF3 and Nuclear Transcription Factor-κB (NF-κB) nuclear translocation. These two transcription factors initiate type I IFN responses, and expression of pro-inflammatory cytokines, respectively (Kim et al., 2023).

### 2.2 Toll like receptor-9

Toll-like receptors (TLRs) are evolutionarily conserved pattern recognition receptors and play a crucial role in innate immune responses, especially the recognition of pathogens in extracellular matrix (Ospelt and Gay, 2010; Takeuchi and Akira, 2010). So far, 10 TLRs (TLR1-TLR10) have been identified in humans. TLRs are classified as a class I integrated transmembrane protein (Bell et al., 2003), The N-terminal domain constitutes the ectodomain, which serves as the recognition site for distinct pathogens-associated molecular patterns (PAMPs) and DAMPs to induce NF-κB activation (Vijay, 2018). TLR-9 is the first TLR proven to recognize DNA, which is mainly located in the endoplasmic reticulum and transported to lysosomes upon activation (Latz et al., 2004; Kim et al., 2008), and recognizes DNA hypomethylated CpG motifs (Barbalat et al., 2011). Nevertheless, TLR-9 is not highly specific for pathogen DNA recognition, and can also be activated by self-DNA (Marshak-Rothstein and Rifkin, 2007). But in any case, the low methylation feature of mitochondrial DNA makes it closer to exogenous DNA and can be recognized by TLR-9 (Bellizzi et al., 2013; Hong et al., 2013). There is still a lack of detailed elucidation on how mitochondrial DNA is transported into lysosomes and recognized by TLR-9, which may be related to mitophagy and MDVs transport (De Leo et al., 2016; Matheoud et al., 2016). TLR9 downstream signaling is transmitted through the adapter Myeloid Differentiation Primary Response Protein 88 (MyD88), which activates Mitogen-activated Protein Kinases and NF-κB to trigger inflammatory responses, or enhance type I IFN responses through IRF7 (Schiller et al., 2012).

### 2.3 Inflammasomes

The activation of inflammasome is an important in innate immune response to PAMPs or DAMPs. The inflammasome is a multi-subunit protein complex composed of receptor proteins, adapter proteins and caspase-1 (Guo et al., 2015). The receptor proteins are responsible for recognizing pathogens or stress signals, and then bind to adapter proteins and recruit procaspase-1 to convert into the active form of caspase-1. Its downstream effects are mainly initiating inflammatory responses by cleaving the N-terminal domain of Gasdermin D, making it an active form that could bind membrane phospholipids to form pores, and cleaving IL-1β and IL-18 precursors (Zheng et al., 2020).

It is known that multiple members of the NOD-like receptor family and PYHIN family can serve as receptor proteins for inflammasomes, including NOD, LRR and Pyrin domain-containing protein 1 (NLRP1), NLRP2, NLRP3, NLRP6, NLRP12, NLR family CARD domain-containing protein 4 (NLRC4), and Absent in Melanoma 2 (AIM2), Interferon-y Inducible Protein 16 (IFI16) (Atianand et al., 2013). Research evidence supports that mtDNA could be recognized as endogenous agonists of inflammasomes, and multiple receptor proteins including NLRP3, NLRC4, and AIM-2 have been reported to recognize mtDNA and activate the inflammasome (Nakahira et al., 2011; Jabir et al., 2015; Zhong et al., 2016). The structure of AIM-2 binding to mtDNA is relatively clear, which has a HIN200 domain at its C-terminus that could recognize and bind double-stranded DNA (Hornung et al., 2009). However, the activation of NLRP3 involves various regulatory factors, including K^+^ efflux, Ca^2+^ signaling, ROS, lysosome rupture (He et al., 2016). Therefore, although the complex of NLRP3 and NLRC4 appears to bind to mtDNA or oxidized mtDNA suggested by co-immunoprecipitation experiments, it is unclear whether they binds directly or other factors are required (West and Shadel, 2017).

## 3 The role of mitochondrial DNA in cardiovascular diseases

### 3.1 Hypertension

According to data from Non-Communicable Disease Risk Factor Collaboration, the age-standardized prevalence of hypertension has exceeded 30% (Zhou et al., 2021). Hypertension increases the risk of cardiovascular events such as coronary heart disease and stroke (Carey et al., 2021). Endothelial dysfunction and vascular structural remodeling is important pathology in hypertension, in which inflammation plays an important role. Recent studies have found that mtDNA levels in the circulation and urine of hypertensive patients are elevated (Eirin et al., 2019), which is related to target organs damage including brain and kidney (Alé et al., 2017; Eirin et al., 2017), and it is proposed that mtDNA involves in the development of vascular pathology and hypertension.

Endothelial cells (ECs) and VSMCs are important components in regulating the contraction and relaxation function and structure of artery. Inflammatory mechanisms are involved in endothelial dysfunction, and mtDNA acts as an important stimuli of inflammatory activation (Simão et al., 2022). Mao Y et al. reported that in palmitic acid treated ECs, the expression levels of pro-inflammatory factors such as MCP1, IFN-γ, IL-1, and adhesion factors ICAM-1 were significantly increased, which enhanced the adhesion of monocytes to ECs. This process is caused by the leakage of mtDNA and activation of the cGAS-STING-IRF3 pathway in ECs. Knockdown of STING attenuated vascular inflammation and macrophage infiltration in high-fat diet fed mice (Mao et al., 2017). They also reported in another study that activation of the cGAS-STING-IRF3 pathway in ECs induced increased MST1 expression, leading to YAP inactivation and nuclear exclusion, thereby inhibiting endothelial cell proliferation, migration and vascular repair (Yuan et al., 2017). Consistent with previous reports, Huang LS et al. found that lipopolysaccharide activated Gasdermin D in ECs and resulted in mitochondrial pores formation and mtDNA leakage. Activated TBK1 phosphorylates LATS1, which subsequently leads to YAP1 degradation and inhibits ECs proliferation and vascular repair (Huang et al., 2020).

**FIGURE 1:**
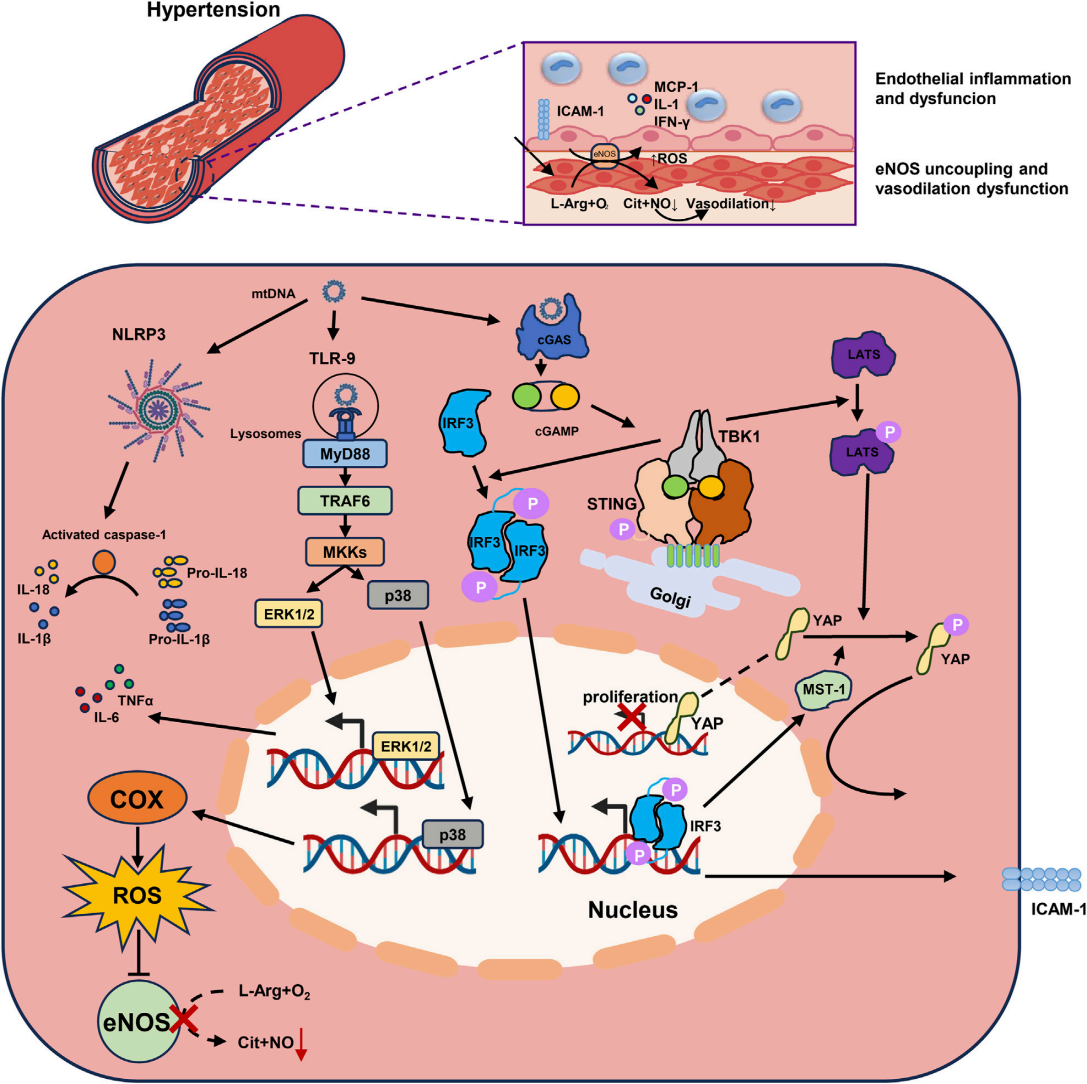
mtDNA promotes endothelial inflammation and dysfunction through multiple inflammatory mechanisms in hypertension. 1) mtDNA activates the cGAS-STING-TBK1 pathway and the downstream IRF3 in endothelial cells. The phosphorylated IRF3 homodimer binds to the ICAM-1 promoter, upregulates the its expression, and enhances adherence of monocytes to endothelial cells. 2) The LATS is also phosphorylated following the activated cGAS-STING-TBK1 pathway, promotes the phosphorylation of YAP and preventing its transport into the nucleus. IRF3 also participate in the phosphorylation of YAP by promoting MST-1 expression, and therefore impaired endothelial cells proliferation. 3) mtDNA transported into lysosomes could be recognized by TLR-9. TLR9 downstream signaling is conducted through the adapter MyD88, which activates ERK1/2 through TRAF6-MKKs to promote the expression of inflammatory factors. MKKs also activates p38 MAPK to promote the expression of COX, which resulting in eNOS uncoupling by generating ROS, and impaired vasodilation. 4) NLRP3 inflammasome in endothelial cells recognizes mtDNA and activates caspase-1 to cleave the precursors of IL-1β and IL-18 into their active forms. mtDNA, Mitochondrial DNA; cGAS, Cyclic GMP-AMP synthase; STING, Stimulator of Interferon Genes; TBK1, TANK Binding Kinase 1; IRF3, Interferon Regulatory Factor 3; ICAM-1, Intercellular Adhesion Molecule-1; LATS, Large tumor suppressor kinase; YAP, Yes-Associated Protein; MST-1, Mammalian Sterile 20-Like Kinase 1; TLR-9, Toll-like Receptors 9; MyD88, Myeloid Differentiation Primary Response Protein 88; ERK1/2, Extracellular signal-regulated kinases 1 and 2; TRAF6, TNF Receptor Associated Factor 6; p38 MAPK, P38 Mitogen Activated Protein Kinase; COX, Cyclo-oxygen-ase; eNOS, Endothelial Nitric Oxide Synthase; ROS, Reactive Oxygen Species; NLRP3, NOD-like receptor thermal protein domain associated protein 3.

In addition, some studies have reported that mtDNA directly impairs endothelial cell-mediated vasodilation. McCarthy CG et al. found elevated circulating mtDNA levels in male spontaneously hypertensive rats (SHR), which were associated with downregulation of mitophagy in the aorta. The TLR-9 inhibitor ODN2088 lowered systolic blood pressure in SHR, while the TLR-9 agonist ODN2395 aggravated hypertension in Wistar-Kyoto rats and SHR rats. It was further found that ODN2395 treatment on mesenteric arteries reduced acetylcholine-induced relaxation and enhanced norepinephrine-induced contraction. This is related to the upregulated COX2 expression rather than the inhibition of eNOS (McCarthy et al., 2015). Consistent with previous reports, Echem C et al. further demonstrated that mtDNA treatment enhanced phenylephrine-induced vasoconstriction in male SHR, while inhibition of TLR-9 by ODN2088 reversed this phenomenon. The expression of IL-6 and TNF-α was also upregulated, whereas the expression and phosphorylation level of eNOS were not affected. Interestingly, Echem C et al. also found that the vasoconstrictive effect of mtDNA on SHR may be affected by gender. mtDNA did not enhance the contraction to phenylephrine of female SHR aorta, which may be related to the reduced phosphorylation level of ERK1/2 (Echem et al., 2019). However, Goulopoulou S et al. reported that mtDNA promotes vasoconstriction and preeclampsia in pregnant rats through TLR-9 and ERK1/2 phosphorylation (Goulopoulou et al., 2012). A recent study obtained umbilical cord serum and tissue from patients with preeclampsia and found that mtDNA leakage from trophoblast cells induced activation of NLRP3/caspase-1/IL-1β signaling in ECs, causing eNOS-related vasodilation dysfunction. The NLRP3 inhibitor INF39 or MCC950 can partially reverse vasodilation dysfunction (Lv et al., 2023) (Summarized in Figure 1).

Excessive proliferation of VSMCs and extracellular matrix synthesis are also important parts in vascular remodeling (Wang et al., 2018; Cai et al., 2021). However, there are few studies on the involvement of mtDNA regulating VSMCs function in hypertension. Arcidiacono MV et al. reported that STING was involved in the osteogenic phenotypic transformation of VSMCs in chronic kidney disease (Arcidiacono et al., 2019). Activation of the cGAS-STING pathway triggers the type I IFN response in VSMCs, resulting in their premature senescence and phenotype switching induced in a paracrine manner (Bi et al., 2021). It has also been reported that TLR-9 could be activated by oxidized hemoglobin-induced lipid peroxidation and leads to the proliferation of pulmonary VSMCs (Loomis et al., 2017). Further research is still required to clarify the role of mtDNA on the phenotype and function of VSMCs in hypertension.

### 3.2 Atherosclerosis

Atherosclerosis is the pathological basis of many cardiovascular diseases such as coronary heart diseases and stroke. Its main feature is lipids depositing in the arterial intima and form into plaques, which leads to reduced blood flow. Plaques rupture can also cause thrombosis and acute complications (Gogulamudi et al., 2023). The pathology of atherosclerosis is complex, including foam cells forming, atherosclerotic plaque forming and rupture, calcification, and thrombus formation. Research evidence has shown that inflammation persist throughout the entire process of atherosclerosis (Wolf and Ley, 2019). mtDNA has also been reported to be involved in the inflammation of atherosclerotic lesions.

In the atherosclerotic lesions of ApoE deficient mice, the cGAMP levels in macrophages was increased and STING was activated, accompanied by the upregulated expression of TNF-α, CCL-2, IFN-β in artery. Bone marrow transplantation after knockout of STING in bone marrow-derived macrophages proved that mtDNA exacerbates the activation of macrophages in atherosclerotic plaques through the cGAS-STING-TBK1 pathway (Pham et al., 2021). Similarly, Liu Y et al. demonstrated that mtDNA induces the inflammatory response of bone marrow-derived macrophages through the STING/NFκB pathway in LDL receptor deficient mice, and proposed that a natural compound Aucubin could inhibit the expression of STING and alleviate atherosclerotic lesions (Liu et al., 2022). Interestingly, Li JL reported that mtDNA-induced inflammation may mediate the association between smoking and atherosclerosis progression. Exposure to e-cigarette smoke in ApoE deficient mice significantly increased mtDNA oxidative damage and upregulated TLR-9 expression in atherosclerotic plaques, as well as subsequent macrophage infiltration and inflammatory cytokines secretion. TLR-9 antagonist could reverse this process (Li et al., 2021). It is also reported that human antimicrobial peptide LL-37 could bind to mtDNA to form a complex, allowing it to escape degradation by DNase II. And the LL-37-mtDNA complex activated the inflammatory response through TLR-9 (Zhang et al., 2015).

**FIGURE 2:**
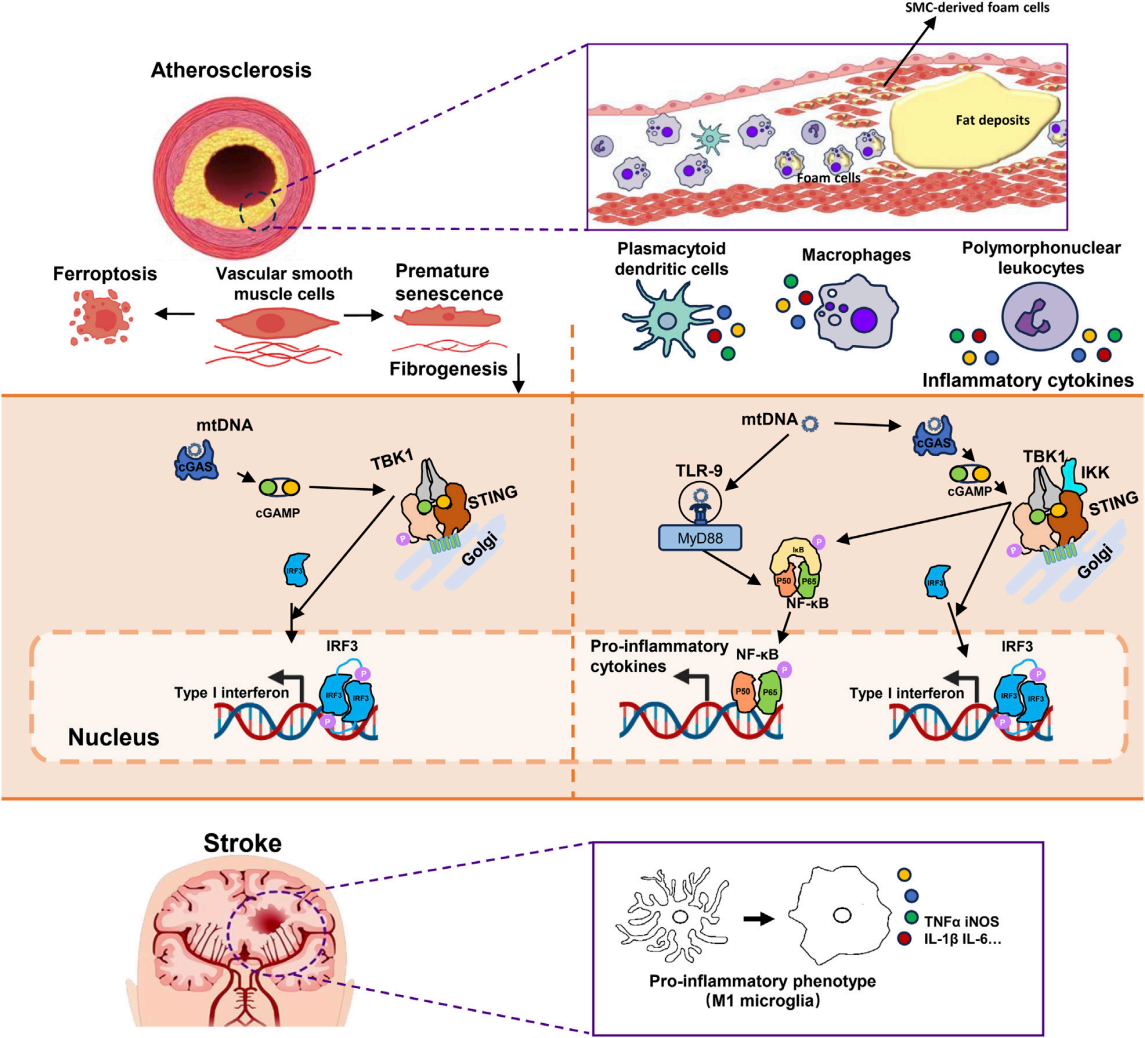
The mechanism of mtDNA promoting plaque vulnerability and inflammation in atherosclerosis and microglial M1 polarization in ischemic stroke. 1) mtDNA participates in the formation of atherosclerosis by affecting the phenotype and function of VSMCs. mtDNA activates the cGAS-STING-IRF3 pathway, triggering type I interferon responses in VSMCs which leads to premature senescence and reduced fibrogenesis ability. It also results in ferritinophagy-dependent ferroptosis of VSMCs and both phenotypes are associated with thinner fibrous caps and contribute to plaque instability. 2) The activation of the cGAS-STING-TBK1 pathway by mtDNA in immune cells leading to increased expression of inflammatory cytokines through both IRF3 and NF-κB. Additionally, NF-κB could also be activated by TLR-9. These processes aggravated the plaque inflammation. 3)Microglia are the main inflammatory cells in the central nervous system. In brain tissue after ischemia-reperfusion injury, mtDNA activates the cGAS-STING pathway in microglia. This activation recruits TBK1 and IKK. Activated TBK1 and IKK subsequently phosphorylate downstream IRF3 and IκBα, respectively. This is followed by the activation of IRF3 and NF-κB, leading to their nuclear translocation and initiating type I interferon responses. These responses can promote M1 polarization of microglia and the expression of pro-inflammatory cytokines. mtDNA, Mitochondrial DNA; VSMCs, Vascular smooth muscle cells; cGAS, Cyclic GMP-AMP synthase; STING, Stimulator of Interferon Genes; IRF3, Interferon Regulatory Factor 3; TBK1, TANK Binding Kinase 1; NF-κB, Nuclear Transcription Factor-κB; TLR-9, Toll-like receptors 9; IKK, Inhibitor of Nuclear Transcription Factor-κB Kinase; IκBα, Inhibitory Subunit of NF-κBα.

In addition, mtDNA could also affects the phenotype and function of VSMCs. Bi XJ et al. reported that oxidized mtDNA in ApoE deficient mouse model of chronic kidney disease triggered type I IFN response in VSMCs through the cGAS-STING pathway, inducing premature senescence and switching from a contractile phenotype to an inflammatory secretory phenotype. This not only aggravates inflammation in atherosclerotic lesions, but also leads to increased plaque vulnerability (Bi et al., 2021). In addition, previous studies reported that lipid-induced programmed cell death such as ferroptosis in VSMCs promoted calcified plaque formation in atherosclerotic lesions (Ma et al., 2021). On this basis, Chen ZD et al. recently reported that mtDNA triggers ferritinophagy-dependent ferroptosis of VSMCs by activating the cGAS-STING pathway. Oleoylethanolamide, an endogenous Peroxisome Proliferator-Activated Receptor α (PPARα) agonist, could attenuate reverse the ferroptosis of VSMCs and arterial intimal calcification (Chen et al., 2023) (Summarized in Figure 2).

### 3.3 Stroke

Stroke is the second leading cause of death worldwide. In 2019, there were 12.2 million incident cases of stroke, and 6.55 million deaths from stroke, accounting for 11.6% of the total deaths (Feigin et al., 2021). Age is one of the most significant risk factors for stroke, and it is reported 12.4% of men and 13.6% of women aged over 80 had stroke (Virani et al., 2021). Atherosclerosis is also an important precursor lesion that causes ischemic stroke, but unlike the situation in hypertension and atherosclerosis with continuous stimulation of external stress, stroke is an acute process of ischemic damage in brain due to cerebrovascular thromboembolism or hemorrhage. Inflammation is also involved in processes such as ischemia/reperfusion injury, blood-brain barrier disruption, and neural regeneration during recovery (Jayaraj et al., 2019).

In the central nervous system, microglia are the main cell type that promotes neuroinflammation. Previous studies have found that a large number of interferon-stimulated genes are upregulated in brain tissue with ischemia-reperfusion injury (McDonough et al., 2017). Studies have found that hypoxia and glucose deficiency can activate type I IFN response, inducing the activation of microglial cells *in vitro* (Minter et al., 2014). Liao, YJ et al. demonstrated that cGAS is activated by mtDNA in microglial cells, activating IRF3 and NF-κB to promote inflammation after cerebral ischemia/reperfusion (Liao et al., 2020). Consistent with previous reports, Jiang GL et al. reported that the cGAS-STING-IRF3 pathway was involved in the M1 polarization of microglia and the secretion of TNF-α in an ischemia-reperfusion model. Knockout of cGAS significantly attenuated microglia-mediated neuroinflammation, inhibited neuronal apoptosis and reduced infarct size (Jiang et al., 2021). Kong LQ et al. also reported that mtDNA leakage after middle cerebral artery occlusion promotes M1 polarization of microglial cells through cGAS-STING signaling, and verified the reduction of cerebral infarct size and recovery of neural function by knockout of STING (Kong et al., 2022) (Summarized in Figure 2).

However, some studies found that AIM2 inflammasomes and cGAS was activated by nuclear DNA in stroke models according to the close colocalization of DAPI and 53BP1 (Li et al., 2020). Gamdzyk M also reported that cytosolic DNA derived from retrotransposon LINE-1 activated cGAS-STING signaling and promoted apoptosis of neurons (Gamdzyk et al., 2020). However, the role of mtDNA in apoptosis and loss of neurons has been rarely reported and remains to be further explored.

## 4 Clinical perspectives and prospect

Cell-free mitochondrial DNA (cf-mtDNA) are considered to be released after cell death or transported through MDVs into circulation. Cosentino N et al. reported that increased cf-mtDNA levels could be detected in more than 91% of 466 patients admitted with confirmed ST-segment elevation myocardial infarction (Cosentino et al., 2021). However, as a non-specific biomarker, cf-mtDNA may be more suitable for screening of metabolic diseases and inflammatory states. Ueda K et al. found that cf-mtDNA levels were higher in smokers, and could predict the risk of atherosclerosis (Ueda et al., 2023). Padilla-Sánchez S D et al. reported that cf-mtDNA levels increased with age and body mass index in healthy adults (Padilla-Sánchez et al., 2020). Alvarado-Vásquez N also reported that cf-mtDNA was associated with endothelial dysfunction in patients with prediabetes (Alvarado-Vásquez, 2015). These suggest the role of cf-mtDNA in reflecting inflammation and metabolic diseases risk.

As for therapeutics, reducing mtDNA leakage appears to be an attractive strategy. Increased burden of senescent cells, which are resistant to apoptosis, is associated with chronic inflammation, and a class of drugs called senolytics could selectively clear senescent cells (Kirkland and Tchkonia, 2020). Iske J et al. recently reported that the senolytics treatment of Dasatinib and Quercetin cleared senescent cells, reduced cf-mtDNA and inhibited inflammation in experimental animals (Iske et al., 2020). In addition, some studies have also raised the possibility of regulating mPTP to inhibit mtDNA leakage. Cyclosporin A has been reported to bind to mitochondrial cyclophilin D to inhibit the opening of mPTP and inhibit the inflammation mediated by mtDNA leakage (Tanveer et al., 1996; Xiao et al., 2018; Liu et al., 2019). Also, substantial efforts have been devoted to the development of compounds that inhibit signaling molecules including cGAS, STING, and TLR-9, and to explore their therapeutic value in inflammatory diseases, which have been detailed elsewhere (Krieg, 2006; Decout et al., 2021).

In summary, mitochondrial dysfunction is a hallmark of cardiovascular aging, and mtDNA leakage is associated with a chronic inflammation and promotes age-related cardiovascular diseases. Reducing the leakage of mtDNA or inhibiting inflammatory signals seems to be attractive therapeutic strategies. The specific mechanisms remain to be further explored to solve the gap in research and development and therapeutic applications.
